# siRNA对血红蛋白H病红系细胞β珠蛋白的调控作用

**DOI:** 10.3760/cma.j.issn.0253-2727.2021.12.009

**Published:** 2021-12

**Authors:** 海灵 程, 容容 刘

**Affiliations:** 广西医科大学第一附属医院血液科，南宁 530021 Department of Hematology, The First Affiliated Hospital of Guangxi Medical University, Nanning 530021, China

**Keywords:** 地中海贫血, 血红蛋白H病, β-珠蛋白, siRNA, Thalassemia, Hemoglobin H disease, β-globin, siRNA

## Abstract

**目的:**

研究靶向siRNA对体外定向分化培养的红系细胞β-珠蛋白的调控作用，为血红蛋白H（Hemoglobin H, HbH）病的基因治疗提供新的理论支持。

**方法:**

①根据β-珠蛋白基因表达结果，在红系细胞中筛选出最佳siRNA序列及其有效作用剂量，检测有效剂量的最佳siRNA对红系细胞β-珠蛋白表达的调控和细胞凋亡的影响。②将有效剂量的最佳siRNA作用于HbH病红系细胞，检测转染后细胞β-珠蛋白表达、活性氧（ROS）和细胞凋亡率，综合评估转染siRNA对HbH病红系细胞的影响。

**结果:**

①siRNA_2_可在转染后96 h内显著下调体外培养的红系细胞β-珠蛋白的表达，但对α-珠蛋白无明显调控作用。siRNA_2_的沉默效应和效应持续时间均与作用剂量有关。②siRNA_2_可下调HbH病红系细胞β-珠蛋白表达，减少细胞内ROS生成，并减少细胞凋亡率。

**结论:**

靶向性siRNA可下调HbH病红系细胞β-珠蛋白表达，减少细胞内ROS产生，下调细胞凋亡率。

## Abstract (en)

**Objective:**

To investigate the regulatory effect of targeted siRNA on β-globin in erythroid cells cultured by targeted differentiation in vitro and provide new theoretical support for gene therapy for hemoglobin H (HbH) disease.

**Methods:**

Based on the β-globin gene expression results, the optimal siRNA sequence and its effective action dose were screened in erythroid cells, and the effect of the effective dose of the optimal siRNA on the regulation of β-globin expression and apoptosis in erythroid cells was examined. The effective dose of the optimal siRNA was applied to erythroid cells with HbH disease. The effects of transfected siRNAs on red line cells with HbH disease were comprehensively evaluated by measuring the expression of β-globin, reactive oxygen species (ROS), and apoptosis rates.

**Results:**

Within 96 hours after transfection, siRNA2 significantly downregulated β-globin expression in in vitro cultured erythroid cells, but not α-globin. siRNA silencing effect and duration of effect were dose-dependent. siRNA2 downregulated β-globin expression, reduced intracellular ROS production, and decreased apoptosis rate in erythroid cells with HbH disease.

**Conclusion:**

Targeted siRNAs can downregulate β-globin expression, reduce intracellular ROS production, and downregulate apoptosis rate in erythroid cells with HbH disease.

血红蛋白H（Hemoglobin H，HbH）病是中间型α-地中海贫血（地贫），主要由于16号染色体上的3个α-基因发生了缺失或突变，从而导致α-珠蛋白合成不足，β-珠蛋白相对过剩，过剩的β链形成HbH。HbH性质不稳定，容易分解为游离β-肽链，在红细胞内沉淀聚集形成H包涵体，对细胞膜造成氧化损伤，改变膜的特性并损害其功能[1]–[2]。已有多个研究显示，减少α-珠蛋白的表达有助于改善β-地贫患者临床症状，安全、可耐受，可长期获益[3]–[5]。然而，关于减少β-珠蛋白在α-地贫中的研究目前尚未检索到相关文献。本研究我们探索siRNA对HbH病红系细胞β-珠蛋白的调控作用，为通过下调β-珠蛋白治疗HbH病提供理论基础。

## 材料与方法

1. 细胞来源与培养：采集健康供者的骨髓液和HbH病患者的骨髓液，标本的采集获我院伦理委员会批准［批件号：2021（KY-E-024）］，经所有研究对象知情同意并签署知情同意书。经磁珠分选CD34^+^造血干细胞，在二阶段培养体系中扩增和定向分化，先在含2 µl/ml StemSpan Erythroid Expansion Supplement及1％青霉素-链霉素的StemSpan SFEM Ⅱ培养基中扩增7 d；后在含3％人AB血清、3 U/ml EPO和1％青霉素-链霉素的StemSpan SFEM Ⅱ培养基中定向分化。

2. 主要试剂：分选磁珠购于德国Miltenyi公司；StemSpan Erythroid Expansion Supplement、StemSpan SFEM Ⅱ培养基购于加拿大Stem cell公司；人AB血清购于美国Gemini Bio公司；siRNA序列由美国Thermo Fisher公司设计与合成（表1），siRNA阴性对照、siRNA荧光对照、活性氧（ROS）试剂盒、Annexin Ⅴ- PI凋亡检测试剂盒购于美国Thermo Fisher公司；电转液与电转杯购于美国BIO-RAD公司；RNA逆转录试剂盒购于日本Takara公司；qPCR试剂盒购于瑞士Roche公司；RNA提取试剂盒购于美国Axygen公司；β-globin抗体、GAPDH抗体购于美国Cell Signaling公司。

**表1:** 转染α-地中海贫血患者红系细胞β-珠蛋白基因的siRNA设计序列

名称	序列
siRNA_1_	5′-GGGCAAGGTGAACGTGGATGAAGTT-3′
siRNA_2_	5′-GGTCTGTGTGCTGGCCCATCACTTT-3′
siRNA_3_	5′-GGCTAATGCCCTGGCCCACAAGTAT-3′

3. siRNA电转染：取定向分化培养后细胞（2～3）×10^6^个，加入200 µl电转染液于0.4 cm电转杯重悬细胞，设置实验组（分别加入siRNA_1_、siRNA_2_、siRNA_3_）、空白对照组（不加入siRNA）、阴性对照组（加入与人类转录序列没有显著相似性的非靶向siRNA-neg）和用于检测转染率的荧光对照组（加入带荧光标记的非靶向siRNA-max），根据组别要求加入siRNA，在220 V、25 ms的条件下进行电转染。

4. RT-PCR检测：收集细胞［每组（2～3）×10^6^］，提取总RNA，参照逆转录试剂盒说明书合成cDNA，在扩增仪进行RT-PCR，反应条件：94 °C预变性5 min，94 °C变性45 s，60 °C退火45 s，72 °C 30 s，共45个循环，最后64～95 °C溶解曲线测定，计算α-、β-珠蛋白基因相对表达。

5. Western bloting检测：收集细胞［每组（2～3）×10^6^］，加入 RIPA细胞裂解液和PMSF提取细胞总蛋白，在梯度浓度为8％～16％的预制胶上以140 V电泳，在4 °C条件下，180 mA电泳转膜至PVDF膜上，快速封闭液封闭15 min，β-globin和GAPDH一抗封闭过夜，二抗室温下孵育1 h，在ChemiDoc™ XRS^+^成像系统中成像，用Image Lab软件分析条带灰度值。

6. 细胞凋亡检测：收集细胞（每组约5×10^5^）200 ×*g*离心5 min，去除培养基。分别在PBS和缓冲液中各洗涤1次，用100 µl缓冲液重悬细胞并加入5 µl Annexin Ⅴ试剂，室温下避光孵育10～15 min，缓冲液中洗涤细胞，200 µl 1×缓冲液重悬细胞，加入5 µl的PI试剂。在4 °C的环境中避光保存与运输，4 h内在流式细胞仪上进行检测分析细胞凋亡率。

7. ROS水平检测：收集细胞200×*g*离心5 min，去除培养基。参照说明书配置ROS工作液，用100 µl ROS工作液重悬各组细胞。在含有5％CO_2_的37 °C培养箱中避光孵育60 min。孵育完毕后，无需洗涤，尽快用流式细胞术分析细胞内ROS水平。

8. 统计分析处理：采用SPSS 23.0进行统计学分析，多组数据比较采用方差分析，组内两两比较采用LSD、Dunnett's T3事后检验，探索不同时间与剂量对基因沉默影响采用多因素方差分析，凋亡与ROS分析采用*t*检验或者Mann-Whitney *U*检验。*P*<0.05为差异有统计学意义。

## 结果

一、调控正常红系细胞β-珠蛋白表达的siRNA筛选

1. 筛选调控β-珠蛋白基因表达的有效siRNA序列：正常红系细胞电转染siRNA_2_后24 h和48 h β珠蛋白基因相对表达量分别为（72±11）％和（73±12）％，与空白对照组比较差异均有统计学意义（*P*＝0.008，*P*＝0.020）。siRNA_1_和siRNA_3_对β-珠蛋白基因表达有也下调作用，但与空白对照组比较差异均无统计学意义（*P*值均>0.05）。转染后24 h和48 h，细胞的α-珠蛋白基因表达水平在各组间差异均无统计学意义（*P*值均>0.05）（图1）。本研究选择siRNA_2_作为调控β-珠蛋白基因的最佳序列，进行下一步实验。

**图1:**
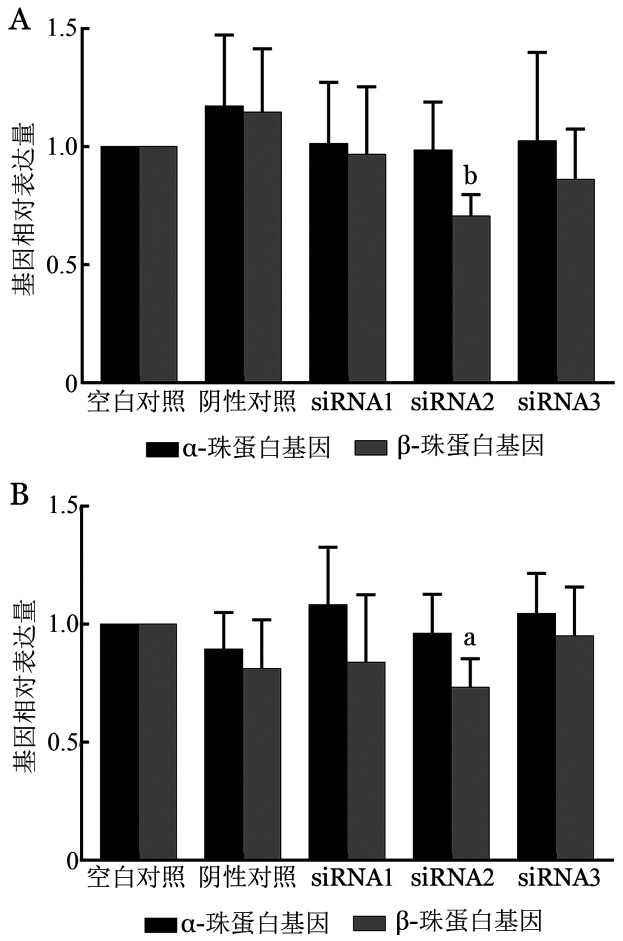
转染不同siRNA后24 h（A）、48 h（B）正常红系细胞α-和β-珠蛋白基因相对表达水平（^a^*P*<0.05，^b^*P*<0.01）

2. 筛选siRNA_2_序列的有效作用剂量：

（1）在基因表达水平上筛选siRNA_2_序列的有效作用剂量：电转染后24 h，4个不同剂量的siRNA_2_对正常红系细胞β珠蛋白基因表达均有显著下调作用［（0.77±0.16）％（*P*＝0.005）、（0.64±0.03）％（*P*<0.001）、（0.63±0.14）％（*P*<0.001）、（0.57±0.05）％（*P*<0.001）］；转染后48 h，150 pmol与200 pmol 的siRNA_2_可显著下调β-珠蛋白基因表达［（0.71±0.15）％（*P*＝0.012）、（0.61±0.18）％（*P*＝0.002）］。转染后72 h和96 h，仅有200 pmol siRNA_2_可显著下调β-珠蛋白基因表达［（0.78±0.07）％（*P*＝0.006）、（0.86±0.03）％（*P*＝0.020）］。与空白对照组比较，以上基因下调差异均有统计学意义。而α-珠蛋白基因表达水平在各浓度组间差异均无统计学意义（*P*值均>0.05）。综上所述，转染48 h内，不同浓度的siRNA_2_均可下调红系细胞β-珠蛋白基因表达；转染后72 h，仅有200 pmol仍维持着沉默效应，且该效应可持续至96 h。可见，高剂量组的沉默效应更显著，持续时间更长。各剂量组对α-珠蛋白基因的表达水平均无下调作用（图2）。

**图2:**
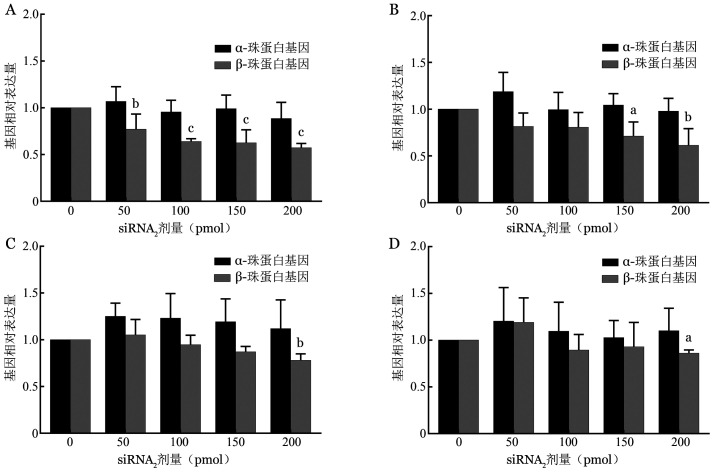
转染siRNA2后24 h（A）、48 h（B）、72 h（C）、96 h（D）正常红系细胞α-和β-珠蛋白基因相对表达水平（^a^*P*<0.05，^b^*P*<0.01，^c^*P*<0.001）

（2）siRNA_2_调控β-珠蛋白基因表达的时间效应：siRNA_2_作用剂量与时间对siRNA_2_沉默效应均有显著影响（*P*值均<0.001），转染后72 h和96 h的β-珠蛋白基因表达水平均显著高于转染后24 h和48 h（*P*值均<0.01），其他剂量组也显示出相同趋势：随着转染后时间的延长，siRNA_2_对β-珠蛋白基因的沉默效应逐渐减弱，甚至消失（图3）。

**图3:**
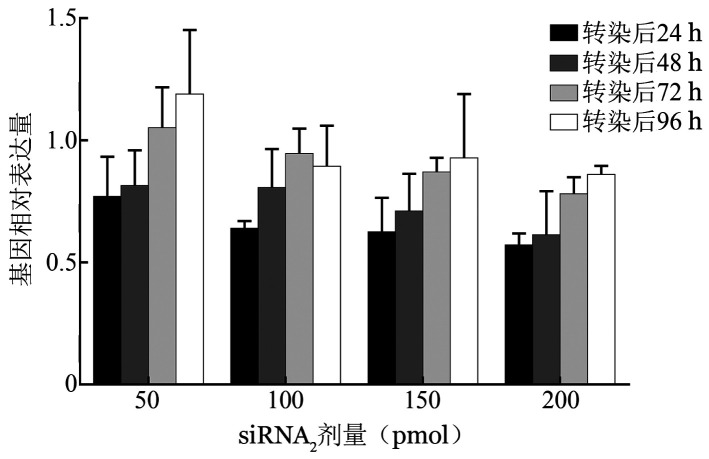
正常红系细胞电转染不同剂量siRNA_2_后不同时间β-珠蛋白基因相对表达水平

3. 有效剂量的siRNA_2_对红系细胞β-珠蛋白表达水平的调控：在转染后48、72、96 h，与阴性对照组和空白对照组相比较，200 pmol组的β-珠蛋白表达水平明显下调，差异均有统计学意义（*P*值均<0.05）。siRNA_2_对β-珠蛋白的下调效应随时间延长逐渐减弱，其作用趋势与β-珠蛋白mRNA表达一致（图4）。

**图4:**
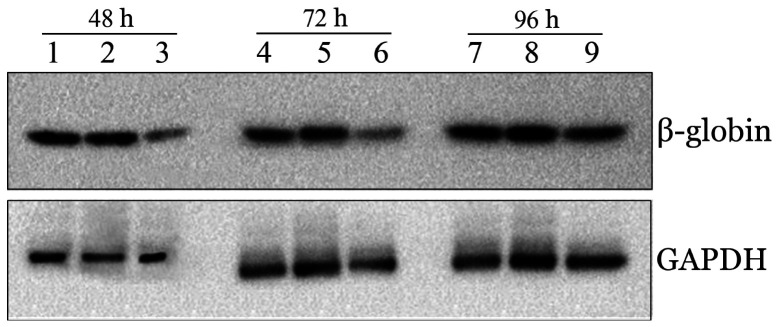
Western blot检测正常红系细胞转染siRNA_2_后不同时间β-珠蛋白的表达水平 1、4、7：空白对照组；2、5、8：200 pmol siRNA-neg组（阴性对照组）；3、6、9：200 pmol siRNA_2_组

4. 有效剂量的siRNA_2_对细胞凋亡率的影响：与空白对照组比较，实验组早期凋亡率均呈轻度增高，差异均无统计学意义（*P*值均>0.05）；而晚期调亡率均出现轻度下调，其中转染后的48 h与72 h凋亡结果，与空白对照组比较，差异均有统计学意义（*P*＝0.039、0.005）。转染后各个时间段细胞的总凋亡率与空白对照组比较差异均无统计学意义（*P*值均>0.05）（图5）。

**图5:**
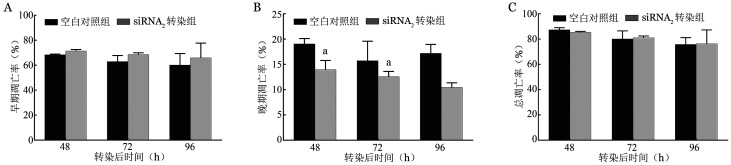
正常红系细胞转染后转染200 pmol siRNA2后不同时间细胞凋亡率（^a^*P*<0.05） A：早期凋亡率；B：晚期凋亡率；C：总凋亡率

二、siRNA_2_对HbH病红系细胞的调控

1. siRNA_2_对HbH病患者红系细胞β-珠蛋白基因表达的调控：转染200 pmol siRNA_2_ 24、48、72 h后，β-珠蛋白基因表达显著下调（*P*＝0.026、0.049、0.034），96 h β-珠蛋白基因表达恢复平衡（图6）。

**图6:**
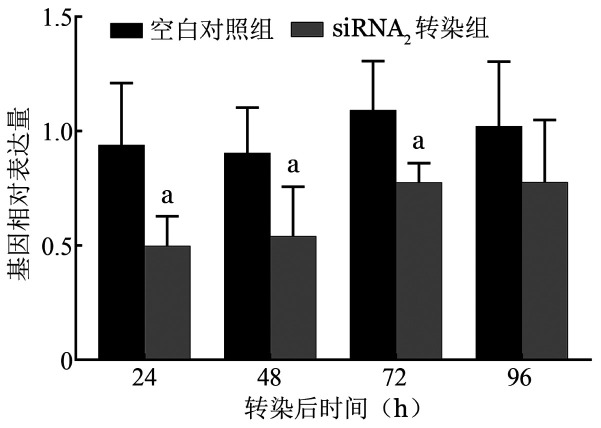
血红蛋白H病红系细胞转染200 pmol siRNA_2_后不同时间β-珠蛋白基因相对表达水平（^a^*P*<0.05）

2. siRNA_2_对HbH红系细胞β-珠蛋白表达的调控：转染后48 h，与空白对照组相比较，200 pmol siRNA_2_组的β-珠蛋白表达水平明显下调（图7），差异均有统计学意义（*P*<0.001）。

**图7:**
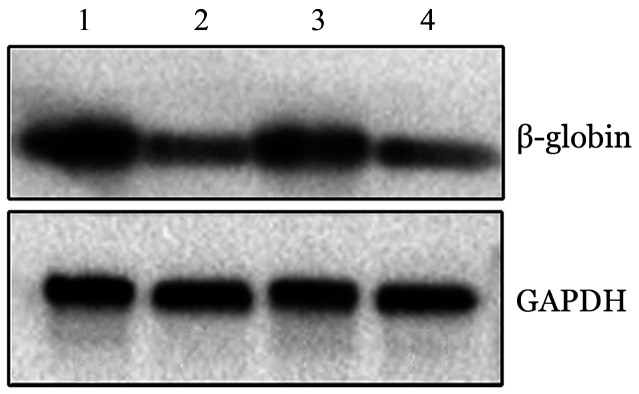
血红蛋白H病红系细胞转染siRNA_2_后48 h β-珠蛋白的表达水平 1、3：空白对照组；2、4：200 pmol siRNA_2_组

3. siRNA_2_对红系细胞内ROS水平的影响：HbH病红系细胞内的ROS水平明显高于正常红系，差异有统计学意义（*P*<0.001）。正常红系细胞转染200 pmol siRNA_2_后细胞内的ROS水平高于空白对照，差异有统计学意义（*P*<0.001）。与之相反，HbH病红系细胞转染200 pmol siRNA_2_后细胞内的ROS水平低于空白对照组，差异有统计学意义（*P*<0.001）。

4. siRNA_2_对HbH红系细胞凋亡的影响：与空白对照比较，除转染后48 h的晚期凋亡率出现增高外，其余实验组细胞的早期凋亡率、晚期凋亡率、总凋亡率均出现下调，且转染后72 h的早期凋亡率与总凋亡率对比空白对照组差异均有统计学意义（*P*＝0.021、0.009）（图8）。

**图8:**
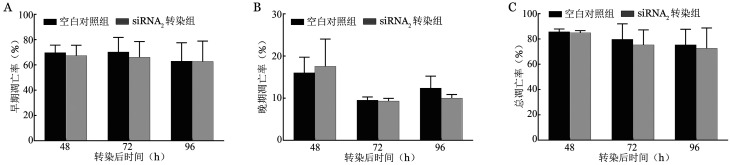
血红蛋白H病红系细胞转染后转染200 pmol siRNA_2_后不同时间细胞凋亡率（^a^*P*<0.05） A：早期凋亡率；B：晚期凋亡率；C：总凋亡率

## 讨论

由于珠蛋白基因缺陷，α/β-珠蛋白链失衡，相对过剩的珠蛋白链沉积于红细胞内，氧化损伤细胞膜和细胞器，阻滞红系成熟并诱导红细胞凋亡[6]–[8]，这与地贫的溶血和无效红细胞生成有关[9]–[10]，减少过剩的β-珠蛋白合成是治疗HbH病溶血的关键。通过RNAi方式减少β-地贫过剩的α-珠蛋白，改善贫血状况的设想已在动物模型中获得验证（表现为HGB升高、RBC增多）[11]–[12]。然而，调控过剩β-珠蛋白肽链在HbH病中的研究目前尚未检索到相关文献。

过去人们对HbH病缺乏足够的关注，近年研究[2],[13]证实，HbH病不只是一种贫血疾病，它更倾向于一种包含贫血、铁过载、肝脾肿大、骨骼畸形、内分泌疾病和心脏疾病等的综合征，各种并发症的发生风险也随着铁在体内的积累而增加，这突出了早期有效干预对非输血依赖地中海贫血（NTDT）患者的必要性[14]。目前，HbH病的治疗方法包括支持治疗、输血治疗、铁螯合治疗、脾切除治疗及必要时的造血干细胞移植[15]。诱导胎儿血红蛋白的药物治疗及探索性的基因治疗应用于β-地贫治疗中[8],[16]。造血干细胞移植仍然是目前治愈地贫的唯一方法[17]，但HLA全相合供者的缺乏、移植排斥反应、移植物抗宿主病（GVHD）及其他与治疗相关毒性的风险，限制着移植的应用。

本实验培养正常人与HbH病患者的红系细胞，通过电穿孔方式转染siRNA，筛选出对细胞β-珠蛋白基因沉默效应最强的siRNA序列及其有效剂量，评估其细胞毒性，并检测红系细胞的ROS水平。证实了靶向β-珠蛋白链mRNA的siRNA_2_对体外定向分化培养的人红系细胞β-珠蛋白有调控作用，siRNA_2_的沉默效应和持续时间与作用剂量有关，且有效剂量的siRNA_2_对细胞凋亡无明显影响，体外培养体系中siRNA_2_沉默效应随着作用时间延长而减弱。筛选获得的有效siRNA在显著下调体外培养的HbH病红系细胞β-珠蛋白的表达水平并减少细胞内ROS产生和细胞凋亡率。siRNA精准靶向目标基因的沉默效应，对恢复α-珠蛋白与β-珠蛋白的平衡有着巨大的潜力，这正是治疗HbH病溶血的关键。

在地贫中，游离的α-或β-珠蛋白链可导致ROS生成增加，ROS攻击细胞膜的磷脂双分子层及膜蛋白结构，引起氧化损伤，损害膜生物的功能，大量的ROS在细胞分化成熟过程中，可能阻滞红系成熟，甚至诱导细胞凋亡，最终加剧了地贫患者外周循环溶血和无效红细胞生成[18]–[21]。Voon等[11]研究显示野生型小鼠红细胞内的ROS明显少于β-地贫模型鼠，而在α-珠蛋白与β-珠蛋白表达相对平衡的复合α-/β-地贫模型小鼠中，红细胞内的ROS与野生型小鼠相近。在本实验中，我们比较正常的红系细胞与HbH病红系细胞ROS水平，结果显示，HbH病红系细胞ROS水平显著高于正常红系，正常红系细胞在转染200 pmol siRNA_2_后，与空白对照组比较，细胞内的ROS水平增高；而HbH病红系细胞转染200 pmol siRNA_2_后，与空白对照组比较，细胞内的ROS水平降低。上述结果与Voon等[11]的结果相符，α-珠蛋白与β-珠蛋白数量失衡与红细胞内ROS水平有关，恢复α-珠蛋白与β-珠蛋白数量平衡可减少细胞内ROS产生，从而减轻地贫患者的外周循环溶血和无效红细胞生成。在本实验中，我们观察到转染siRNA_2_后HbH红系细胞β-珠蛋白表达减少，细胞内ROS水平下降，细胞凋亡率下调。然而细胞凋亡率下调较小，这可能与电穿孔对细胞造成的损伤有关，且细胞的体外培养难以真实地模拟体内环境，具体的机制尚有待进一步研究。

与基因编辑相比，siRNA有方便、灵活、低脱靶风险的优点，目前在多领域治疗研究中取得了不错的进展，一些基于siRNA干预的治疗方法已经进入了临床研究[22]–[25]，如与年龄相关的黄斑变性、糖尿病性黄斑水肿、呼吸道病毒感染、人类免疫缺陷病毒感染和多种肿瘤。2018年8月10日，siRNA药物Onpattro（Patisiran）获得了第一份全球批准用于治疗成人由遗传性甲状腺素转运蛋白介导的淀粉样变性（hATTR）引起的多发性神经疾病。随后被欧盟药品管理局（EMA）批准用于治疗成人hATTR 1或2期多发性神经疾病。这是首个被批准上市siRNA药物，具有里程碑式的意义，为siRNA应用带来了新的曙光。对于NTDT患者来说值得期待。

综上，本研究证实了siRNA可下调HbH病红系细胞β-珠蛋白的表达并减少细胞内ROS的产生和细胞凋亡率，有望成为治疗HbH病的新途径。然而，本研究也存在不足之处，siRNA _2_对HbH病红系细胞凋亡率的下调幅度较小，考虑这与电转染对细胞的损伤有关，下一步将通过建立HbH病的鼠模型进行体内实验，进一步探究siRNA的有效性与安全性。
